# Exclusion of sulfide:quinone oxidoreductase from mitochondria causes Leigh-like disease in mice by impairing sulfide metabolism

**DOI:** 10.1172/JCI170994

**Published:** 2024-06-13

**Authors:** Eiki Kanemaru, Kakeru Shimoda, Eizo Marutani, Masanobu Morita, Maria Miranda, Yusuke Miyazaki, Claire Sinow, Rohit Sharma, Fangcong Dong, Donald B. Bloch, Takaaki Akaike, Fumito Ichinose

**Affiliations:** 1 Anesthesia Center for Critical Care Research, Department of Anesthesia, Critical Care and Pain Medicine, Massachusetts General Hospital and Harvard Medical School, Boston, Massachusetts, USA.; 2 Department of Environmental Medicine and Molecular Toxicology, Tohoku University Graduate School of Medicine, Sendai, Japan.; 3 Howard Hughes Medical Institute, Department of Molecular Biology, Massachusetts General Hospital and Harvard Medical School, Boston, Massachusetts, USA.; 4 Broad Institute of MIT and Harvard, Cambridge, Massachusetts, USA.; 5 Department of Systems Biology, Harvard Medical School, Boston, Massachusetts, USA.; 6 Department of Medicine, Division of Rheumatology, Allergy and Immunology, Massachusetts General Hospital, Boston, Massachusetts, USA.

**Keywords:** Metabolism, Therapeutics, Mitochondria, Mouse models, Neurological disorders

## Abstract

Leigh syndrome is the most common inherited mitochondrial disease in children and is often fatal within the first few years of life. In 2020, mutations in the gene encoding sulfide:quinone oxidoreductase (SQOR), a mitochondrial protein, were identified as a cause of Leigh syndrome. Here, we report that mice with a mutation in the gene encoding SQOR (Sqor^ΔN/ΔN^ mice), which prevented SQOR from entering mitochondria, had clinical and pathological manifestations of Leigh syndrome. Sqor^ΔN/ΔN^ mice had increased blood lactate levels that were associated with markedly decreased complex IV activity and increased hydrogen sulfide (H_2_S) levels. Because H_2_S is produced by both gut microbiota and host tissue, we tested whether metronidazole (a broad-spectrum antibiotic) or a sulfur-restricted diet rescues Sqor^ΔN/ΔN^ mice from developing Leigh syndrome. Daily treatment with metronidazole alleviated increased H_2_S levels, normalized complex IV activity and blood lactate levels, and prolonged the survival of Sqor^ΔN/ΔN^ mice. Similarly, a sulfur-restricted diet normalized blood lactate levels and inhibited the development of Leigh syndrome. Taken together, these observations suggest that mitochondrial SQOR is essential to prevent systemic accumulation of H_2_S. Metronidazole administration and a sulfur-restricted diet may be therapeutic approaches to treatment of patients with Leigh syndrome caused by mutations in *SQOR*.

## Abstract 2

Pharmacological or dietary inhibition of H2S production prolonged survival in a preclinical model of Leigh syndrome caused by the *SQOR* gene mutation.

## Introduction

Leigh syndrome is an inherited metabolic disorder that affects the central nervous system and is the most common mitochondrial disease in children. The genetic abnormalities in Leigh syndrome are remarkably diverse, and mutations in more than 80 genes have been reported to cause the disease. The majority of these genes produce proteins that are involved in the generation of energy in mitochondria (1, 2). Patients with Leigh syndrome appear healthy at birth, but subsequently experience developmental delay, ataxia, hypotonia, dystonia, and seizures. Clinical manifestations may be triggered by fasting or infection, usually in the first year of life, and children typically die within 2–3 years (1, 3). The neurological decline is associated with the development of bilateral, symmetrical lesions in the brain stem and basal ganglia with marked gliosis (1). Patients have evidence of abnormal energy metabolism, characterized by increased levels of lactic acid.

Hydrogen sulfide (H_2_S) is produced by various natural and industrial sources and is generally considered a highly toxic substance for aerobic organisms because it inhibits mitochondrial complex IV activity (4). However, H_2_S also has a number of physiological functions and enhances mitochondrial respiration as an electron donor (4, 5). In mammalian cells, H_2_S is synthesized from cysteine and homocysteine in reactions catalyzed by cystathionine β-synthase, cystathionine gamma-lyase, and 3-mercaptopyruvate sulfurtransferase. The gut microbiome was shown to be a major source of H_2_S, as H_2_S levels were significantly reduced in the plasma, feces, and gastrointestinal tissues of germ-free mice (6, 7). H_2_S is catabolized in mitochondria by sulfide oxidation enzymes. Conversion of H_2_S to glutathione persulfide by sulfide:quinone oxidoreductase (SQOR) in mitochondria is the first step in the process of sulfide oxidation, which donates electrons to mitochondrial complex III via coenzyme Q (8). Glutathione persulfide is oxidized to form sulfite by persulfide dioxygenase (ETHE1), and sulfite is further oxidized to form sulfate and thiosulfate by sulfite oxidase and thiosulfate sulfurtransferase, respectively (4).

In 2020, three patients with Leigh syndrome were found to have mutations in the gene encoding SQOR (9). These patients had very low levels of SQOR protein and markedly reduced SQOR enzyme activity in muscle and liver. Complex IV activity in muscle and liver was reduced, despite normal levels of complex IV proteins. The children appeared to be healthy at birth, but developed encephalopathy and metabolic acidosis, with increased levels of lactic acid, after episodes of gastroenteritis and prolonged fasting. Magnetic resonance imaging (MRI) revealed T2-hyperintense lesions in the basal ganglia and brain stem. Two of the patients died at ages 4 and 8, respectively, while the third patient survived despite recurrent episodes of encephalopathy. Because H_2_S inhibits mitochondrial complex IV activity, and the ability to catabolize H_2_S is likely to be impaired as a result of severely depressed SQOR protein levels, the authors hypothesized that increased levels of H_2_S may be contributing to the development of Leigh disease in these patients (9). However, whether the accumulation of H_2_S, caused by loss-of-function mutations in *SQOR*, inhibits mitochondrial complex IV activity and impairs systemic energy metabolism has not been established.

Previous studies showed that strategies to decrease H_2_S production were beneficial in patients with ethylmalonic encephalopathy, a subtype of Leigh syndrome that is caused by ETHE1 deficiency (10). The pathogenesis of ethylmalonic encephalopathy involves inhibition of complex IV activity by H_2_S accumulation (10). Modulation of H_2_S-producing gut bacteria by metronidazole is the primary treatment for ethylmalonic encephalopathy (1, 11). In addition, a sulfur-restricted diet improved biochemical parameters and clinical outcome in a child with ethylmalonic encephalopathy (12).

The current study was designed to elucidate the pathogenetic mechanisms responsible for Leigh syndrome induced by mutations in the gene encoding SQOR and explore possible therapies. For these studies, we used a mouse model, in which the gene encoding SQOR is replaced by a gene that lacks the sequence encoding the N-terminal mitochondrial localization sequence (SQORΔN) (13). Here, we report that Sqor^ΔN/ΔN^ mice, in which SQOR is excluded from mitochondria, recapitulate the clinical characteristics of Leigh syndrome, including progressive neurological deficits, brain lesions, and shortened lifespan. Sqor^ΔN/ΔN^ mice have increased blood lactate levels that are associated with markedly decreased complex IV activity and increased H_2_S levels. Reduction of H_2_S production, either by treatment with metronidazole or by a sulfur-restricted diet, was associated with the prevention of Sqor^ΔN/ΔN^ mice from developing neurological symptoms and abnormal energy metabolism. These results demonstrate the essential role of mitochondrial SQOR in preventing systemic H_2_S accumulation, and suggest a potential approach to the treatment of Leigh syndrome caused by SQOR gene mutations.

## Results

### Clinical manifestations of mitochondrial SQOR deficiency in Sqor^ΔN/ΔN^ mice.

**Figure 1:**
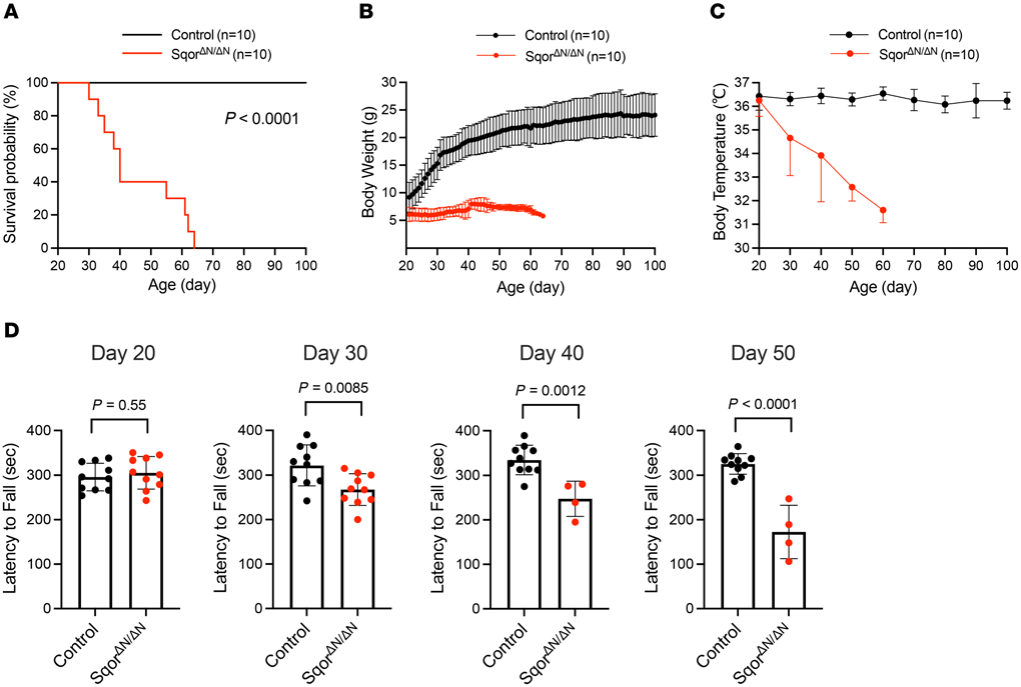
Sqor^ΔN/ΔN^ mice had decreased weight gain, progressive hypothermia, motor dysfunction, and shortened lifespan. (**A**) Kaplan-Meier survival probability curve for Sqor^ΔN/ΔN^ and control mice. Log-rank *P* value and sample size are shown. (**B**) Body weight trajectory of Sqor^ΔN/ΔN^ and control mice. *n* = 10 mice for each group. Data are presented as means with SD. (**C**) Body temperature trajectory of Sqor^ΔN/ΔN^ and control mice. *n* = 10 mice for each group. Data are presented as means with SD. (**D**) Time to fall from rotarod in Sqor^ΔN/ΔN^ and control mice at postnatal ages 20, 30, 40, and 50 days. Comparisons were made using unpaired 2-tailed *t* test. *n* = 4 to 10 mice for each group. Data are presented as means with SD.

To investigate the consequences of mitochondrial SQOR deficiency, we measured survival, body weight, body temperature, and locomotor activity in Sqor^ΔN/ΔN^ mice. Because WT and Sqor^ΔN/+^ mice appeared to be phenotypically normal, both WT and Sqor^ΔN/+^ mice were used as controls in subsequent experiments. The median survival of Sqor^ΔN/ΔN^ mice was significantly shorter than that of control mice (Sqor^ΔN/ΔN^ vs. control, 40 vs. undetermined [longer than at least 210 days]; *P* < 0.0001 by log-rank test; Figure 1A). Sqor^ΔN/ΔN^ mice gradually became emaciated and developed ataxia with a wide-based gait and seizures. Although Sqor^ΔN/ΔN^ mice were indistinguishable from WT and heterozygous (Sqor^ΔN/+^) littermates at birth, the rate of growth of Sqor^ΔN/ΔN^ mice decreased beginning at 3 weeks, around the time of weaning. Sqor^ΔN/ΔN^ mice were smaller than control mice beginning at 3 weeks of age, and their body weight never exceeded 10 g (Figure 1B). The average body temperature of Sqor^ΔN/ΔN^ mice decreased with age, reaching 32°C shortly before their death, whereas the body temperature of control mice ranged between 36°C and 37°C (Figure 1C).

The rotarod test, which measures the ability to maintain grip strength, balance, and resist fatigue on an accelerating rotating rod, was used to evaluate motor function of Sqor^ΔN/ΔN^ mice. Control mice were able to balance on the rotarod for approximately 300 seconds. In contrast, the ability of Sqor^ΔN/ΔN^ mice to balance on the rotarod decreased gradually over time (Figure 1D). Taken together, the results show that Sqor^ΔN/ΔN^ mice have decreased weight gain, progressive hypothermia, neurological deficits, and shortened lifespan.

### Sqor^ΔN/ΔN^ mice had decreased complex IV activity, increased systemic H_2_S levels, and an impaired metabolic status.

**Figure 2:**
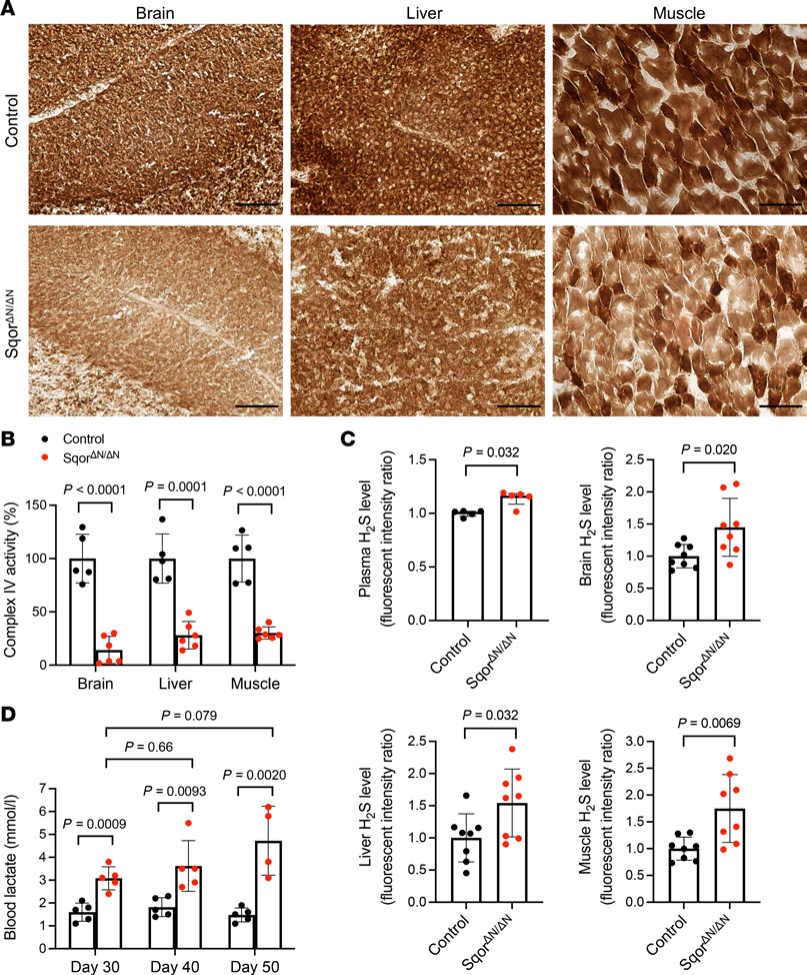
Sqor^ΔN/ΔN^ mice had decreased complex IV activity, increased systemic H_2_S levels, and an impaired metabolic status. (**A**) Histochemical staining was used to measure cytochrome *c* oxidase (COX) activity in Sqor^ΔN/ΔN^ and control mice. Control mouse brain, liver, and muscle sections are stained dark brown; the same tissues from Sqor^ΔN/ΔN^ mice are light brown, indicating decreased complex IV activity. Scale bars: 100 μm. (**B**) Complex IV activity in isolated mitochondria of the brain, liver, and muscle. The mean values of complex IV activity in control mice were set to 100%. Comparisons were made using unpaired 2-tailed *t* test. *n* = 5–6 mice for each group. Data are presented as means with SD. (**C**) Sulfide levels in plasma, brain, liver, and muscle were measured using HSip-1. The sulfide levels in Sqor^ΔN/ΔN^ mice were compared with those in control mice (the sulfide level for control mice was set to 1). Data were analyzed using Mann-Whitney test for plasma, and unpaired 2-tailed *t* test for brain, liver, and muscle. *n* = 5–8 mice for each group. Data are presented as medians with interquartile range for plasma, and as means with SD for brain, liver, and muscle. (**D**) Compared with control mice, the blood lactate levels in Sqor^ΔN/ΔN^ mice were higher at postnatal ages 30, 40, and 50 days. Comparisons between Sqor^ΔN/ΔN^ and control mice at postnatal ages 30, 40, and 50 days were made using unpaired 2-tailed *t* test. Mixed-effects analysis with Dunnett’s multiple-comparison test was performed to compare the blood lactate levels at postnatal ages 30, 40, and 50 days in Sqor^ΔN/ΔN^ mice. *n* = 4–5 mice for each group. Data are presented as means with SD.

To investigate the pathogenesis of the neurological dysfunction, emaciation, hypothermia, and shortened lifespan in Sqor^ΔN/ΔN^ mice, we evaluated complex IV activity and levels of tissue H_2_S and blood lactate. We used cytochrome *c* oxidase (COX) histochemical staining to evaluate complex IV activity in Sqor^ΔN/ΔN^ mice. COX histochemical staining assesses complex IV activity by the intensity of brown staining produced by the indamine polymer that is a product of COX-catalyzed oxidation of 3,3′-diaminobenzidine (14). In control mice, using COX histochemical staining, sections from brain, liver, and muscle appeared dark brown (Figure 2A). In contrast, brain, liver, and muscle sections from Sqor^ΔN/ΔN^ mice were light brown, indicating decreased complex IV activity (Figure 2A). To quantify complex IV activity in Sqor^ΔN/ΔN^ mice, we performed a complex IV activity assay, which measures the oxidation of reduced cytochrome *c*. The complex IV activity in the brain, liver, and muscle from Sqor^ΔN/ΔN^ mice was significantly decreased compared with that in tissues from control mice (14.3% ± 12.9%, 28.0% ± 12.9%, and 30.2% ± 5.8% of control in brain, liver, and muscle, respectively; Figure 2B). These results show that Sqor^ΔN/ΔN^ mice have severely decreased complex IV activity in the brain, liver, and muscle.

To investigate whether the loss of mitochondrial SQOR increases H_2_S levels in the tissues of Sqor^ΔN/ΔN^ mice, we measured relative H_2_S levels in plasma, brain, liver, and muscle using the fluorescence probe HSip-1. When H_2_S binds to HSip-1, the intensity of emitted fluorescence increases (15). The H_2_S levels in plasma, brain, liver, and muscle from Sqor^ΔN/ΔN^ mice were significantly higher than the levels in control mice (Figure 2C). These results suggest that the loss of mitochondrial SQOR increases systemic H_2_S levels in Sqor^ΔN/ΔN^ mice.

Patients with Leigh syndrome have abnormal energy metabolism, indicated by the presence of lactic acidosis (9). To determine whether Sqor^ΔN/ΔN^ mice also have abnormal energy metabolism, we measured blood lactate levels in Sqor^ΔN/ΔN^ and control mice. The blood lactate levels in Sqor^ΔN/ΔN^ mice were significantly higher than those in control mice at postnatal ages 30, 40, and 50 days (Figure 2D). These results suggest that Sqor^ΔN/ΔN^ mice have increased blood lactate levels, which is one of the diagnostic criteria for Leigh syndrome.

Excessive accumulation of H_2_S can increase levels of C4 and C5 acylcarnitines (10) and thiosulfate (16), changes that have been observed in a mouse model of ethylmalonic encephalopathy (ETHE1-knockout mice) (10). To further characterize the effects of excess H_2_S levels in Sqor^ΔN/ΔN^ mice and examine the biochemical similarity of Sqor^ΔN/ΔN^ mice to ETHE1-knockout mice, we measured C4 and C5 acylcarnitines in plasma and thiosulfate in the brain stem and liver of Sqor^ΔN/ΔN^ mice using liquid chromatography–tandem mass spectrometry (LC-MS/MS) and gas chromatography–tandem mass spectrometry (GC-MS/MS). As was reported in ETHE1-knockout mice, the level of thiosulfate was increased in the brain stem of Sqor^ΔN/ΔN^ mice, compared with control animals. However, in contrast to ETHE1-knockout mice, the level of thiosulfate in the liver and the levels of C4 and C5 acylcarnitines in plasma did not differ between Sqor^ΔN/ΔN^ mice and control mice (Supplemental Figure 1; supplemental material available online with this article; https://doi.org/10.1172/JCI170994DS1). These results suggest that Sqor^ΔN/ΔN^ mice exhibit limited similarity to ETHE1-knockout mice. Nonetheless, these results further provide indirect evidence of H_2_S accumulation in the brains of Sqor^ΔN/ΔN^ mice.

### Sqor^ΔN/ΔN^ mice showed integrated stress response and NADH-reductive stress.

The integrated stress response (ISR) is an evolutionarily conserved pathway in eukaryotic cells, which restores cellular homeostasis in response to various stresses, including mitochondrial dysfunction, nutrient deficiency, unfolded protein stress, and infection (17, 18). A previous study showed that dysfunction of the mitochondrial electron transport chain, induced by impaired NADH oxidation, triggered the ISR in myoblasts (18). Activating transcription factor 4 (ATF4) is the ISR master regulator, and DNA damage–inducible transcript 3 protein (DDIT3) is a key component of the ISR. Activation of ATF4 and DDIT3 stimulates the expression of 2 additional components of the ISR, growth/differentiation factor 15 (GDF15) and fibroblast growth factor 21 (FGF21). Increased levels of ATF4, DDIT3, GDF15, and FGF21 have been recognized as promising blood biomarkers for human mitochondrial disorders (18–23). To investigate whether the ISR is induced in Sqor^ΔN/ΔN^ mice, we measured the messenger RNA (mRNA) levels of ATF4, DDIT3, GDF15, and FGF21 at postnatal age 40 days. Compared with control mice, Sqor^ΔN/ΔN^ mice had a marked increase in the levels of mRNAs, encoding all 4 proteins, in liver and muscle (Supplemental Figure 2), suggesting that the ISR was triggered in Sqor^ΔN/ΔN^ mice.

To investigate whether the biomarkers associated with NADH-reductive stress increase in Sqor^ΔN/ΔN^ mice (24), we performed a metabolomic analysis using plasma from Sqor^ΔN/ΔN^ and control mice at postnatal age 40 days. The level of biomarkers associated with NADH-reductive stress, including the ratios of lactate to pyruvate and α-hydroxybutyrate to α-ketobutyrate, were significantly higher in Sqor^ΔN/ΔN^ mice than control mice (Supplemental Figure 3), suggesting that the NADH-reductive stress was induced by the mutation in *SQOR*.

### Sqor^ΔN/ΔN^ mice had lesions in basal ganglia, midbrain, and brain stem.

**Figure 3:**
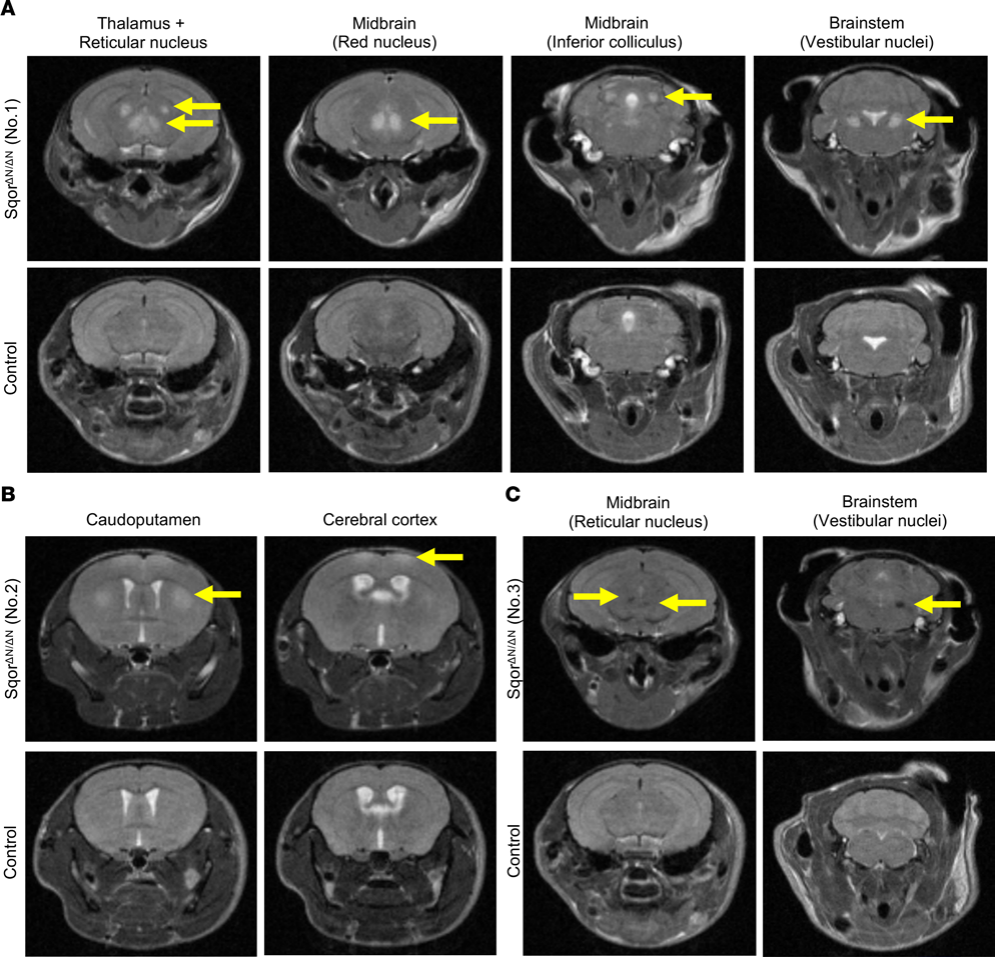
Sqor^ΔN/ΔN^ mice had lesions in basal ganglia, midbrain, and brain stem. T2-weighted brain MRI was performed on three 8-week-old Sqor^ΔN/ΔN^ mice: Sqor^ΔN/ΔN^ mouse no. 1 (**A**), Sqor^ΔN/ΔN^ mouse no. 2 (**B**), and Sqor^ΔN/ΔN^ mouse no. 3 (**C**). Brain lesions in Sqor^ΔN/ΔN^ mice are indicated by yellow arrows. Brain MRI scans of a control mouse, at the same anatomical levels as those of Sqor^ΔN/ΔN^ mice, are shown as reference in the bottom panels of **A**, **B**, and **C**.

Patients with Leigh syndrome caused by mutations in the gene encoding SQOR had T2-hyperintense bilateral, symmetrical lesions in the basal ganglia, thalamus, and hippocampus, as well as unilateral lesions in the cerebral cortex (9). To investigate whether Sqor^ΔN/ΔN^ mice also develop brain lesions, we performed T2-weighted brain MRI on 3 Sqor^ΔN/ΔN^ mice and 1 control mouse. The first Sqor^ΔN/ΔN^ mouse had bilateral, symmetrical hyperintense lesions in the thalamus, reticular nucleus, red nucleus, inferior colliculus, and vestibular nuclei (Figure 3A). The second Sqor^ΔN/ΔN^ mouse had bilateral, symmetrical hyperintense lesions in the caudoputamen and cerebral cortex (Figure 3B). The third Sqor^ΔN/ΔN^ mouse had numerous small, round hypointense lesions in the reticular nucleus and vestibular nuclei (Figure 3C). No lesions were detected in the brain of the control mouse. These results show that Sqor^ΔN/ΔN^ mice have brain abnormalities similar to those seen in patients with Leigh syndrome caused by mutations in the gene encoding SQOR.

### Sqor^ΔN/ΔN^ mice had neurodegeneration with gliosis and acute and subacute hemorrhage.

**Figure 4:**
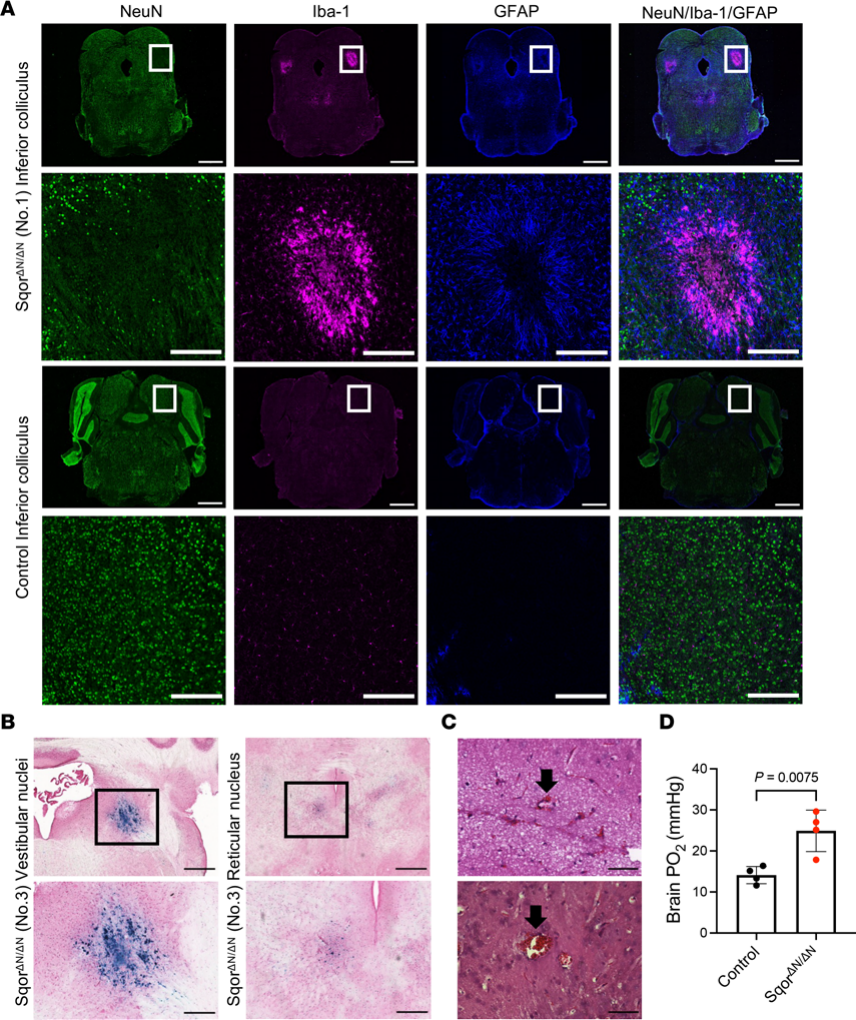
Sqor^ΔN/ΔN^ mice had neurodegeneration with gliosis, acute and subacute hemorrhage, and brain tissue hyperoxia. (**A**) The portions of the inferior colliculus of Sqor^ΔN/ΔN^ mouse no. 1 that were indicated in the brain MRI scans were used for immunohistochemical staining (upper panels). Staining for NeuN, Iba-1, and GFAP was performed to evaluate neurodegeneration and gliosis. The corresponding portions of the inferior colliculus of a control mouse were used for control staining (lower panels). An enlargement of the boxed regions is provided below each cross section. Scale bars: 1,000 μm in low-magnification images and 250 μm in high-magnification images. (**B**) Photomicrographs of brain lesions in vestibular nuclei and reticular nucleus from Sqor^ΔN/ΔN^ mouse no. 3. Prussian blue staining was used to detect subacute hemorrhage. Blue stain indicates the location of previous episodes of bleeding. An enlargement of the boxed regions is provided below each cross section. Scale bars: 500 μm in low-magnification images and 200 μm in high-magnification images. (**C**) Photomicrographs of fresh hemorrhages surrounding small vessels in the white matter. Hematoxylin and eosin staining was performed to detect acute hemorrhage. Fresh hemorrhages surrounding small vessels are indicated by black arrows. Scale bars: 50 μm. (**D**) Brain partial pressure of oxygen was measured using an optical oxygen probe at the reticular nucleus and caudoputamen of Sqor^ΔN/ΔN^ mice and control mice at postnatal age 40 days. Comparisons were made using unpaired 2-tailed *t* test. *n* = 4 mice for each group. Data are presented as means with SD. GFAP, glial fibrillary acidic protein; Iba-1, ionized calcium-binding adaptor molecule 1; NeuN, neuronal nuclei; PO_2_, partial pressure of oxygen.

To investigate the pathological features of the brain abnormalities seen on MRI imaging of Sqor^ΔN/ΔN^ mouse brains, we stained brain sections for the presence of neuronal nuclei antigen (NeuN), ionized calcium-binding adaptor molecule 1 (Iba-1), and glial fibrillary acidic protein (GFAP). Antibodies directed against NeuN identify mature neuronal cell bodies in tissue sections (25). Increased expression of Iba-1 and of GFAP is a marker for activation of microglial cells and astrocytes, respectively (26, 27). In coronal brain sections from Sqor^ΔN/ΔN^ mouse no. 1, there was a loss of NeuN-positive neurons and an increase in Iba-1–positive cells in the lesion core, and an increase in GFAP-positive cells in the lesion border in the inferior colliculus (Figure 4A), thalamus, reticular nucleus, red nucleus, and vestibular nuclei (Supplemental Figure 4). Similar lesions were observed in the caudoputamen of Sqor^ΔN/ΔN^ mouse no. 2 (Supplemental Figure 5). These results in Sqor^ΔN/ΔN^ mice, including neuronal loss and gliosis with prominent microglia and astrocyte activation in the brain lesions, are similar to the pathological features seen in patients with Leigh syndrome.

Prussian blue staining was used to further investigate the pathological features of brain lesions identified by T2-weighted MRI hypointensities in Sqor^ΔN/ΔN^ mice. Prussian blue detects iron in hemosiderin, which is a marker of subacute hemorrhage (28). Blue staining was observed in tissue sections prepared from portions of the brain that were identified by hypointense lesions on T2-weighted MRI (Figure 3C), indicating previous episodes of bleeding. Prussian blue staining of the vestibular nuclei and reticular nucleus of Sqor^ΔN/ΔN^ mouse no. 3 is shown in Figure 4B. We used hematoxylin and eosin staining (29) to demonstrate that Sqor^ΔN/ΔN^ mice had acute perivascular microhemorrhages in cerebral white matter (Figure 4C). Taken together, the results show that Sqor^ΔN/ΔN^ mice have evidence of acute and subacute hemorrhages in the brain.

### Brain hyperoxia in Sqor^ΔN/ΔN^ mice.

Approximately 90% of all oxygen consumed by the body is used by complex IV of the electron transport chain in a reaction that reduces oxygen, in the presence of hydrogen, to produce water (30). In patients with mitochondrial disease, the rate of oxygen consumption in the brain is decreased (31). Because complex IV activity was severely reduced with the accumulation of H_2_S in Sqor^ΔN/ΔN^ mice, we hypothesized that oxygen consumption is impaired in Sqor^ΔN/ΔN^ mice, leading to brain tissue hyperoxia. To determine the oxygen level in brain tissue of Sqor^ΔN/ΔN^ mice, we used an optical oxygen probe (32) to measure the partial pressure of oxygen (PO_2_) at the reticular nucleus and caudoputamen, where brain lesions were observed in MRI. Brain tissue PO_2_ in Sqor^ΔN/ΔN^ mice was significantly higher than that in control mice (Sqor^ΔN/ΔN^ vs. control, 24.9 ± 5.0 vs. 14.1 ± 2.1 mmHg; *P* = 0.0075; Figure 4D). The observed brain tissue hyperoxia is consistent with inhibition of complex IV activity by increased H_2_S levels in Sqor^ΔN/ΔN^ mice.

### Metronidazole decreased the systemic H_2_S levels, alleviated Leigh-like disease, and prolonged the lifespan of Sqor^ΔN/ΔN^ mice.

**Figure 5:**
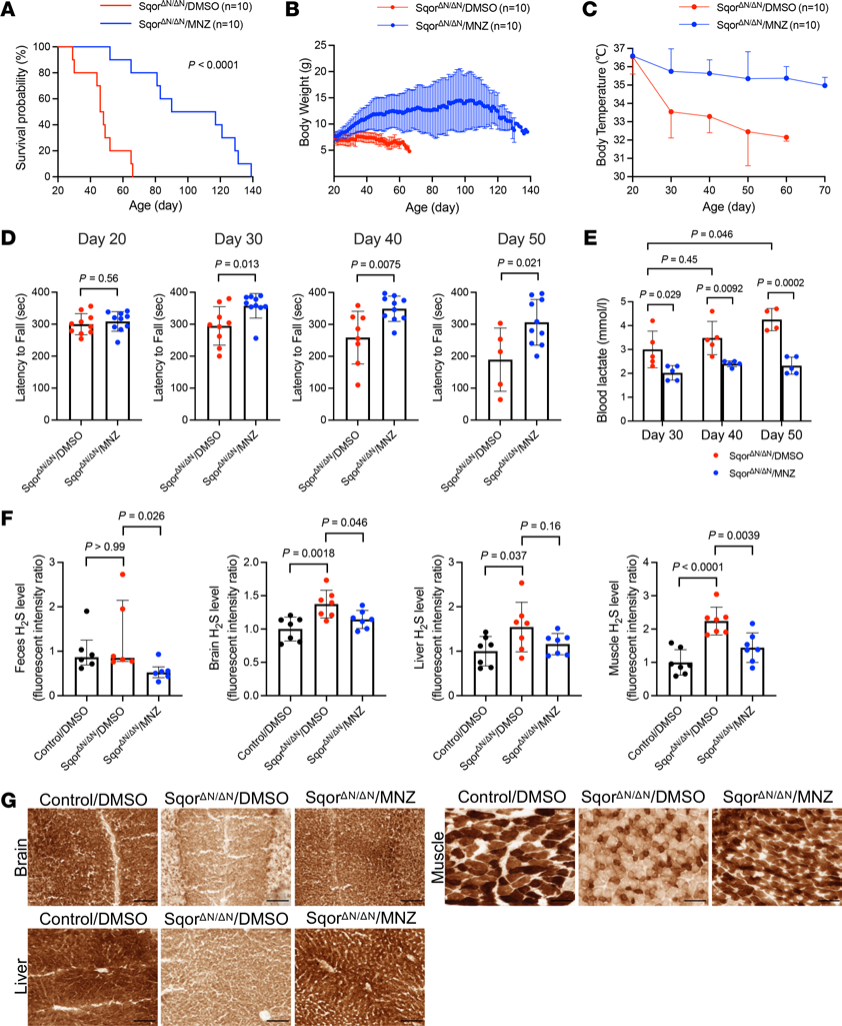
Metronidazole decreased systemic H_2_S levels, alleviated Leigh-like disease, and prolonged the lifespan of Sqor^ΔN/ΔN^ mice. (**A**) Kaplan-Meier survival probability curve of Sqor^ΔN/ΔN^ mice treated with metronidazole or DMSO (4.4%). Log-rank *P* value and sample size are shown. (**B**) Body weight trajectory of Sqor^ΔN/ΔN^ mice treated with metronidazole or DMSO. *n* = 10 mice for each group. (**C**) Body temperature trajectory of Sqor^ΔN/ΔN^ mice treated with metronidazole or DMSO. *n* = 10 mice for each group. (**D**) Results of the rotarod test at postnatal ages 20, 30, 40, and 50 days in Sqor^ΔN/ΔN^ mice treated with metronidazole or DMSO. Two-tailed *t* test. *n* = 5–10 mice for each group. (**E**) Blood lactate levels at postnatal ages 30, 40, and 50 days in Sqor^ΔN/ΔN^ mice treated with metronidazole or DMSO. Comparisons between Sqor^ΔN/ΔN^ mice treated with metronidazole or DMSO were made using unpaired 2-tailed *t* test. Mixed-effects analysis with Dunnett’s multiple-comparison test was performed to compare blood lactate levels at postnatal ages. *n* = 4–5 mice for each group. (**F**) Sulfide levels in feces, brain, liver, and muscle in Sqor^ΔN/ΔN^ mice treated with metronidazole or DMSO and control mice treated with DMSO (the sulfide level for control mice treated with DMSO was set to 1). Data were analyzed using Kruskal-Wallis test with Dunn’s multiple-comparison test for feces, and 1-way ANOVA with Dunnett’s multiple-comparison test for brain, liver, and muscle. *n* = 6–7 mice for each group. Data are presented as medians with interquartile range for feces. (**G**) Histochemical staining was used to assess COX activity in a Sqor^ΔN/ΔN^ mouse treated with metronidazole or DMSO and a control mouse treated with DMSO. Brown color indicates cytochrome *c* oxidase activity levels. Scale bars: 100 μm. MNZ, metronidazole. Data are presented as means with SD unless indicated otherwise.

The gut microbiome is a major source of H_2_S in mammals (6, 7), and the broad-spectrum antibiotic metronidazole can reduce the number of H_2_S-producing bacteria in the gastrointestinal tract (11). We hypothesized that treatment with metronidazole might inhibit the development of Leigh-like disease in Sqor^ΔN/ΔN^ mice by reducing H_2_S-producing gut bacteria. To consider this possibility, metronidazole (at 30 mg/kg of body weight) was administered twice a day by intraperitoneal injection to 10 Sqor^ΔN/ΔN^ mice (Sqor^ΔN/ΔN^/MNZ group) beginning 21 days after birth. Because metronidazole was dissolved in 4.4% dimethylsulfoxide (DMSO) diluted with distilled water, 10 control Sqor^ΔN/ΔN^ mice (Sqor^ΔN/ΔN^/DMSO group) were similarly treated with 4.4% DMSO twice a day. Metronidazole extended the median survival of Sqor^ΔN/ΔN^ mice (Sqor^ΔN/ΔN^/MNZ vs. Sqor^ΔN/ΔN^/DMSO, 104 vs. 47 days; *P* < 0.0001 by log-rank test; Figure 5A). Treatment with metronidazole enabled Sqor^ΔN/ΔN^ mice to gain weight and prevented progressive hypothermia (Figure 5, B and C). In contrast to the control group, Sqor^ΔN/ΔN^/MNZ mice were able to maintain motor coordination and balance, as assessed using the rotarod test (Figure 5D). In addition, while the blood lactate levels increased in Sqor^ΔN/ΔN^/DMSO mice, treatment with metronidazole prevented hyperlactacidemia in Sqor^ΔN/ΔN^ mice (Figure 5E). Furthermore, the biomarkers associated with NADH-reductive stress, including the ratios of lactate to pyruvate and α-hydroxybutyrate to α-ketobutyrate, were increased in DMSO-treated Sqor^ΔN/ΔN^ mice compared with DMSO-treated control mice at postnatal age 40 days (Supplemental Figure 3). While treatment with metronidazole tended to attenuate the increase in the lactate/pyruvate ratio (*P* = 0.0552 vs. DMSO-treated Sqor^ΔN/ΔN^ mice), it did not attenuate the increase of the α-hydroxybutyrate/α-ketobutyrate ratio in SQOR^ΔN/ΔN^ mice at postnatal age 40 days. These results indicate that Sqor^ΔN/ΔN^ mice exhibit NADH-reductive stress, which was partially reversed by metronidazole (Supplemental Figure 3).

To explore the mechanism by which metronidazole inhibited the development of Leigh-like disease in Sqor^ΔN/ΔN^ mice, we measured the levels of H_2_S in feces, brain, liver, and muscle using HSip-1. To confirm that HSip-1 is a valid method to measure the levels of H_2_S in tissue homogenates and feces, we measured fluorescence intensity in tissues and feces with exogenously added sodium sulfide (Na_2_S). Increasing amounts of Na_2_S produced a dose-dependent increase in HSip-1–associated fluorescence intensity (Supplemental Figure 6). At baseline, there was no difference in H_2_S levels in feces between control mice and Sqor^ΔN/ΔN^ mice treated with DMSO, suggesting that the mutation does not alter the levels of H_2_S produced by gut flora (Figure 5F). In the brain, liver, and muscle, H_2_S levels were significantly higher in DMSO-treated Sqor^ΔN/ΔN^ mice than in DMSO-treated control mice (control/DMSO) (Figure 5F). Treatment with metronidazole reduced the H_2_S levels in the feces, brain, liver, and muscle of Sqor^ΔN/ΔN^ mice (Figure 5F). Treatment with metronidazole also restored complex IV activity in the brain, liver, and muscle of Sqor^ΔN/ΔN^ mice, as assessed using COX histochemical staining (Figure 5G). These results suggest that metronidazole decreased H_2_S levels in host tissues, restored complex IV activity, and prolonged the lifespan of Sqor^ΔN/ΔN^ mice.

### A sulfur-restricted diet decreased the systemic H_2_S levels, alleviated the clinical manifestations, and prolonged the lifespan of Sqor^ΔN/ΔN^ mice.

**Figure 6:**
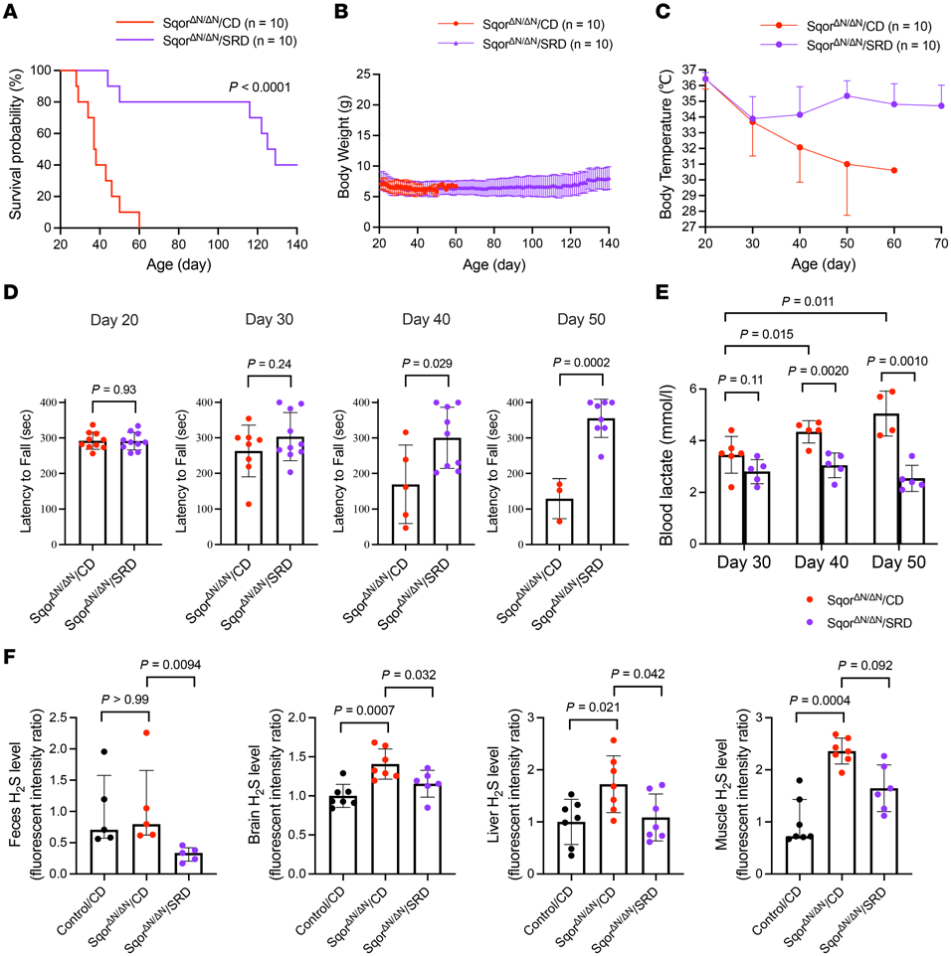
A sulfur-restricted diet decreased the systemic H_2_S levels, alleviated the clinical manifestations, and prolonged the lifespan of Sqor^ΔN/ΔN^ mice. (**A**) Kaplan-Meier survival probability curve was used to quantify increased survival of Sqor^ΔN/ΔN^ mice fed a sulfur-restricted diet compared with mice fed a control diet. Log-rank *P* value and sample size are shown. (**B**) Body weight trajectory of Sqor^ΔN/ΔN^ mice fed a sulfur-restricted diet or a control diet. *n* = 10 mice for each group. Data are presented as means with SD. (**C**) Body temperature trajectory of Sqor^ΔN/ΔN^ mice fed a sulfur-restricted diet or a control diet. *n* = 10 mice for each group. Data are presented as means with SD. (**D**) A rotarod was used to measure motor function of Sqor^ΔN/ΔN^ mice fed a sulfur-restricted diet or a control diet at postnatal ages 20, 30, 40, and 50 days. Comparisons were made using unpaired 2-tailed *t* test. *n* = 3–10 mice for each group. Data are presented as means with SD. (**E**) Compared with Sqor^ΔN/ΔN^ mice fed a control diet, blood lactate levels in Sqor^ΔN/ΔN^ mice fed a sulfur-restricted diet were lower at postnatal ages 40 and 50 days. Comparisons between Sqor^ΔN/ΔN^ mice fed a sulfur-restricted diet or a control diet were made using unpaired 2-tailed *t* test. Mixed-effects analysis with Dunnett’s multiple-comparison test was performed to compare blood lactate levels at postnatal ages 30, 40, and 50 days. *n* = 4–6 mice for each group. Data are presented as means with SD. (**F**) Sulfide levels in feces, brain, liver, and muscle were measured using HSip-1. Sulfide levels in Sqor^ΔN/ΔN^ mice fed a sulfur-restricted diet or a control diet were compared with those in control mice fed a control diet (the sulfide level for control mice fed a control diet was set to 1). Data were analyzed using Kruskal-Wallis test with Dunn’s multiple-comparison test for feces and muscle, and 1-way ANOVA with Dunnett’s multiple-comparison test for brain and liver. *n* = 5–7 mice for each group. Data are presented as medians with interquartile range for feces and muscle, and as means with SD for brain and liver. CD, control diet; SRD, sulfur-restricted diet.

To further investigate the effect of decreased H_2_S levels on the development of Leigh-like disease in Sqor^ΔN/ΔN^ mice, we examined the effects of a sulfur-restricted diet. Decreased ingestion of cystine and methionine would be expected to decrease both endogenous (host) and exogenous (microbiome) production of H_2_S (7). Beginning at age 21 days, 10 Sqor^ΔN/ΔN^ mice in the treatment arm were fed a sulfur-restricted diet (Sqor^ΔN/ΔN^/SRD), which did not contain cystine and contained only 0.1% methionine per total diet weight. Ten Sqor^ΔN/ΔN^ mice in the control arm received a control diet (Sqor^ΔN/ΔN^/CD), which contained the same nutritional composition as the sulfur-restricted diet but included 0.4% cystine and 0.5% methionine per total diet weight. Although the sulfur-restricted diet extended the median survival of Sqor^ΔN/ΔN^ mice (Sqor^ΔN/ΔN^/SRD vs. Sqor^ΔN/ΔN^/CD, 127 vs. 38 days; *P* < 0.0001 by log-rank test; Figure 6A), it did not restore the normal increase in body weight over time (Figure 6B). The sulfur-restricted diet prevented the progression of hypothermia after postnatal day 30 (Figure 6C). As in metronidazole-treated Sqor^ΔN/ΔN^ mice, the sulfur-restricted diet prevented the development of motor dysfunction, which was observed in the control-fed Sqor^ΔN/ΔN^ mice, as assessed using the rotarod test (Figure 6D). The blood lactate levels gradually increased in the control-fed Sqor^ΔN/ΔN^ mice, but the sulfur-restricted diet prevented hyperlactacidemia in Sqor^ΔN/ΔN^ mice (Figure 6E). The beneficial effects of the sulfur-restricted diet were associated with reduced H_2_S levels in feces, brain, liver, and muscle of Sqor^ΔN/ΔN^ mice (Figure 6F). Taken together, the results further support the pathogenetic role of H_2_S accumulation in Leigh-like disease in Sqor^ΔN/ΔN^ mice and suggest a therapeutic strategy for patients with Leigh syndrome caused by SQOR gene mutations.

## Discussion

In this study, we used a mouse model to investigate the biochemical and developmental consequences of a loss-of-function mutation in the gene encoding SQOR. In addition, we used this model to investigate two strategies to potentially treat patients with Leigh syndrome caused by pathogenic SQOR gene mutations. Compared with control animals, Sqor^ΔN/ΔN^ mice had decreased weight gain, neurological deficits, and decreased lifespan. The mutation in Sqor^ΔN/ΔN^ mice induced the ISR and the NADH-reductive stress response, which are induced by mitochondrial dysfunction. In addition, Sqor^ΔN/ΔN^ mice developed bilateral, symmetrical, brain lesions in the basal ganglia, midbrain, and brain stem, which were characterized by neuronal loss with prominent microglia and astrocyte activation, and both acute and subacute hemorrhages. The loss of mitochondrial SQOR was associated with a marked reduction in complex IV activity in brain, liver, and muscle that was accompanied by brain tissue hyperoxia and progressive, systemic hyperlactacidemia.

Sqor^ΔN/ΔN^ mice had increased H_2_S levels in the plasma, brain, liver, and muscle. Prevention, or delay, of the clinical manifestations of Leigh syndrome in Sqor^ΔN/ΔN^ mice, either by treatment with the broad-spectrum antibiotic metronidazole or by feeding of a sulfur-restricted diet, was associated with decreased H_2_S levels in these tissues. Importantly, these observations suggest that pharmacological or dietary inhibition of H_2_S production by microbiota and host tissues may be a promising treatment for patients with Leigh syndrome caused by SQOR gene mutation.

The lack of SQOR in mouse mitochondria allows excessive accumulation of H_2_S in host cells, inhibits complex IV activity, and leads to abnormal energy metabolism. Brain tissue hyperoxia observed in Sqor^ΔN/ΔN^ mice is similar to the phenotype of mice that are deficient in the mitochondrial complex I subunit NADH:ubiquinone oxidoreductase subunit S4 (NDUFS4), another mouse model of Leigh syndrome (32, 33). Together with the reported phenotype of ETHE1-knockout mice, these observations suggest that the mitochondrial sulfide oxidation pathway, as opposed to other non-canonical sulfide oxidation mechanisms (34–36), plays the predominant role in limiting systemic H_2_S accumulation. The reduction in H_2_S levels in feces and host tissues and the reversal of complex IV inhibition by treatment with metronidazole suggest that the gut microbiome is a major source of H_2_S in Sqor^ΔN/ΔN^ mice. H_2_S is produced by various gut bacteria, including γ-proteobacteria such as sulfate- and sulfite-reducing bacteria and Clostridia species (37), which are sensitive to metronidazole (11). The results presented here are consistent with a previous paper by Shen and colleagues, which reported that the levels of free H_2_S and bound sulfane sulfur were decreased by 60% and 50%–80%, respectively, in germ-free mice (6).

*Enterococcus*, Enterobacteriaceae, and Clostridia species degrade sulfur-containing amino acids, including cysteine and methionine, using desulfhydrases, in a reaction that produces H_2_S in the intestine (38). A previous report showed that fecal sulfide concentrations were correlated with the amount of dietary animal proteins (39). In addition, Flannigan and colleagues reported that when colonic tissue or feces were grown in media that lacked both methionine and l-cysteine, both colonic cells and bacteria were unable to produce H_2_S, suggesting that H_2_S production from host tissues and feces depends on the availability of these amino acids (7). Consistent with these previous reports, in the current study, a sulfur-restricted diet reduced the H_2_S levels in the feces, brain, liver, and muscle of Sqor^ΔN/ΔN^ mice.

In the recent report of 3 patients with Leigh syndrome caused by mutations in *SQOR*, the patients became symptomatic after gastrointestinal infections and prolonged fasting (9). These conditions would be expected to increase H_2_S production from enhanced protein catabolism. In the absence of infection or fasting, the patients were reported to be generally healthy, and they were relatively asymptomatic for several years (9). There are similarities between the disease presentation in patients and the phenotype of Sqor^ΔN/ΔN^ mice. As previously reported, Sqor^ΔN/ΔN^ mice were born normally according to the predicted Mendelian ratio and were indistinguishable from WT and Sqor^ΔN/+^ littermates before weaning (13). Growth of Sqor^ΔN/ΔN^ mice ceased around the weaning period, and Sqor^ΔN/ΔN^ mice gradually became emaciated, developed ataxia, and died within 10 weeks of age (13). Sqor^ΔN/ΔN^ mice became symptomatic as they acquired gut microbiome in the mouse colony. The coprophagy of mice may have contributed to the relatively rapid and progressive deterioration of Sqor^ΔN/ΔN^ mice.

The Leigh syndrome associated with SQOR gene mutations has some similarities with ethylmalonic encephalopathy. As was observed in ETHE1-knockout mice, Sqor^ΔN/ΔN^ mice had increased levels of H_2_S and inhibition of complex IV activity in multiple organs (10). Giordano and colleagues reported that patients with ethylmalonic encephalopathy had diffuse vascular damage with morphological features of acute and subacute hemorrhages in the brain and colonic mucosa (40). Similar pathological changes were observed in ETHE1-knockout mice. The authors hypothesized that diffuse vascular damage was a unique pathogenic mechanism in ethylmalonic encephalopathy because high levels of circulating H_2_S were toxic to endothelial cells (40). In the current study, Sqor^ΔN/ΔN^ mice exhibited acute and subacute hemorrhages in the brain, supporting this hypothesis.

Although increased blood levels of C4 and C5 acylcarnitines and thiosulfate are hallmarks of ethylmalonic encephalopathy, C4 and C5 acylcarnitines were not increased in plasma, and thiosulfate was increased only in the brain but not in the liver, of Sqor^ΔN/ΔN^ mice. Nonetheless, the same treatment for patients with ethylmalonic encephalopathy prevented the clinical manifestations of Leigh syndrome in Sqor^ΔN/ΔN^ mice. The pharmacological or dietary inhibition of H_2_S production by microbiota and host tissues appears to be a rational treatment strategy in diseases associated with excessive H_2_S accumulation in the body.

Although the sulfur-restricted diet prolonged the lifespan of Sqor^ΔN/ΔN^ mice, it did not restore the normal increase in body weight over time. This result is consistent with previous reports that investigated the effects of the sulfur-containing amino acid restriction on the gut microbiome in rodents (41). Nichenametla and colleagues reported that the mechanism by which the sulfur-restricted diet causes the decrease in body weight gain differed depending on the age at which the sulfur-restricted diet was started (42). In young rodents, sulfur restriction was associated with the growth inhibition, including a decrease in insulin-like growth factor, which may contribute to the decrease in body weight gain (42). Because Sqor^ΔN/ΔN^ mice were fed the sulfur-restricted diet beginning at age 21 days, the poor body weight gain in the Sqor^ΔN/ΔN^ mice fed the sulfur-restricted diet may be due to the mechanism described by Nichenametla and colleagues. A previous study reported that the beneficial effects of dietary restriction on longevity in 8- to 10-week-old adult mice were associated with increased H_2_S production via enhancement of the transsulfuration pathway, which appears to contradict the results of this study (43). The age-dependent differences in the effects of diet restriction may explain the discrepancy between the current and the previous report regarding the effects of dietary restriction on H_2_S production (44).

A potential limitation of this study is that the genetic mutation in Sqor^ΔN/ΔN^ mice (homozygotes for 14-bp deletion of CCTGGTGATGGCCC at exon 2 of the SQOR gene) differed from that in Leigh syndrome patients (homozygotes for the pathogenic variant c.637G>A or c.446delT) (9, 13). In the murine model, SQOR is excluded from mitochondria but is expressed, in a truncated form, in the cytosol of Sqor^ΔN/ΔN^ mice (13). In contrast, in patients with Leigh syndrome caused by SQOR gene mutation, protein levels are markedly decreased in the mitochondria and cytoplasm of all cells (9). However, because catabolism of H_2_S requires localization of SQOR to mitochondria (8), the genetic mutation in Sqor^ΔN/ΔN^ mice severely impaired the ability of cells to catabolize H_2_S, similar to the condition in patients. Notably, the phenotype of SQOR-knockout mice is very similar to that of Sqor^ΔN/ΔN^ mice (45, 46). Future studies will be required to confirm the therapeutic effects of metronidazole and sulfur-restricted diet in SQOR-knockout mice.

In conclusion, the current study revealed that mice in which SQOR is excluded from mitochondria recapitulate the clinical characteristics of patients with Leigh syndrome caused by SQOR gene mutations, including brain lesions, progressive motor dysfunction, and shortened lifespan. Sqor^ΔN/ΔN^ mice exhibit hyperlactacidemia, which is associated with systemic accumulation of H_2_S and markedly decreased complex IV activity. Treatment with metronidazole or feeding of a sulfur-restricted diet prevented or delayed the clinical manifestations of Leigh syndrome in Sqor^ΔN/ΔN^ mice. Although H_2_S can be oxidized by other non-canonical mechanisms, these observations illuminate the essential and predominant role of SQOR in preventing systemic H_2_S accumulation. Our results suggest therapeutic strategies for the treatment of Leigh syndrome caused by mutations in the gene encoding SQOR.

## Methods

### Sex as a biological variable.

Both male and female mice were used in this study in almost equal proportions. Therefore, in this study, sex was not considered a biological variable.

### Animal care.

The study design and the description of experiments followed the Animal Research: Reporting of *In Vivo* Experiments (ARRIVE) guidelines. Sqor^ΔN/ΔN^ mice were generated as previously reported (13). Male and female Sqor^ΔN/+^ mice were crossed to obtain Sqor^ΔN/ΔN^ and control mice. Pups were genotyped and weaned at 20 days after birth. All mice were housed in a temperature- and humidity-controlled room with a 12-hour light/12-hour dark cycle, and were given food, water, and hydrated gel ad libitum. Body weights were recorded every day, and rectal body temperatures were measured every 10 days starting at 20 days after birth. When more than two Sqor^ΔN/ΔN^ pups were born in a litter, Sqor^ΔN/ΔN^ pups were equally allocated to treatment group and control group to minimize the variation in phenotype between different littermates. Mice were randomly assigned to different treatments within each pair after pairing of mice based on sex and body weight.

### Sulfur-restricted diet and control diet.

The sulfur-restricted diet (A22052003, Research Diets) lacked cystine and contained only 0.1% methionine per total diet weight. The control diet (A19032701, Research Diets) contained the same nutritional composition as the sulfur-restricted diet but included 0.4% cystine and 0.5% methionine per total diet weight.

### Behavior test.

The amount of time that mice were able to balance on an accelerating rotarod (Ugo Basile, Stoelting Co.), “latency to fall,” was measured at postnatal ages 20, 30, 40, and 50 days. The parameters of the rotarod were set as follows: initial speed of 5 rpm, maximum speed of 40 rpm, and maximum time of 400 seconds. The test was performed 3 times, with a 10-minute break between sessions to allow for recovery. The median latency to fall was reported to avoid the incorporation of aberrant behavior trials. If a mouse latched on to the rod rather than walked on the rod for more than 3 rotations, the mouse was considered to have fallen.

### Cytochrome c oxidase histochemical staining.

Immediately after mice were sacrificed by isoflurane overdose (5%) followed by decapitation, brain, liver, and muscle were collected and frozen on dry ice (14). The tissues were sectioned to 20 μm thickness using a cryotome (CM1850UV, Leica Biosystems). Cytochrome *c* oxidase (COX) activity was evaluated using the COX incubation solution of COX/Succinate Dehydrogenase Double Histochemistry Stain Kit (VB-3022, VitroVivo Biotech). The stained sections were examined using an epifluorescence microscope (Nikon Eclipse 80i, Nikon Instruments Inc.). Two biological replicates were performed for each organ.

### H&E, Prussian blue, and immunohistochemical staining.

For Prussian blue and immunohistochemical staining, mice were deeply anesthetized with isoflurane and transcardially perfused with ice-cold PBS, followed by 4% paraformaldehyde. Tissues were sectioned at 25 μm using a cryotome (CM3050S, Leica Biosystems). For hematoxylin and eosin (H&E) staining, mice were sacrificed by isoflurane overdose (5%) followed by decapitation, tissues were fixed in 4% paraformaldehyde, and 20 μm sections were prepared.

For immunohistochemical staining, sections were incubated at 4°C for 48 hours with the following primary antibodies diluted in double-salt PBS (2.8 mM NaH_2_PO_4_ • 2H_2_O, 7.2 mM Na_2_HPO_4_, 300 mM NaCl) containing 0.3% Triton X-100 (Sigma-Aldrich): mouse anti–neuronal nuclei antiserum (NeuN; 1:500 dilution; MCA1B7, EnCor Biotechnology), rabbit anti–ionized calcium-binding adaptor molecule 1 antiserum (Iba-1; 1:500 dilution; 019-19741, Fujifilm Wako Chemicals), and goat anti–glial fibrillary acidic protein antiserum (GFAP; 1:500 dilution; ab53554, Abcam). After 3 washes with double-salt PBS, sections were incubated for 1 hour at room temperature with Alexa Fluor 488–conjugated donkey anti-mouse antiserum (A32766), Alexa Fluor 555–conjugated donkey anti-rabbit antiserum (A32794), and Alexa Fluor 647–conjugated donkey anti-goat antiserum (A21447) (all from Thermo Fisher Scientific). Secondary antisera were obtained from Invitrogen (Thermo Fisher Scientific) and were diluted 1:500 in double-salt PBS containing Triton X-100 (0.3%). The sections were stained with DAPI (ab228549, Abcam) before mounting with Fluoromount-G medium (0100-01, Southern Biotech). The stained sections were imaged with a confocal microscope (LSM 800, Carl Zeiss Meditec AG) using an EC Plan-Neofluar ×10/0.30 NA M27 objective lens in tile-scan mode. A montage of single tiles was performed using the stitching algorithm of Zen Blue 2.6 software (Carl Zeiss Meditec AG). Fiji ImageJ (version 2.0.0-rc-69/1.52p) was used to generate maximum-intensity projections, remove background, and uniformly adjust brightness and contrast (47).

Prussian blue and H&E staining were performed using the Prussian Blue Stain kit (ab150674, Abcam) and H&E Stain Kit (VB-3000, VitroVivo Biotech) following the manufacturer’s instructions. The stained sections were examined using a Nikon Eclipse 80i microscope.

### Measurements of gene expression.

The mRNA levels associated with the ISR were measured as previously described using liver and muscle (48). Quantitative real-time PCR was performed using TaqMan Fast Advanced Master Mix (Applied Biosystems) with primers targeting ATF4 (Mm00515325_g1), DDIT3 (Mm01135937_g1), GDF15 (Mm00442228_m1), FGF21 (Mm07297622_g1), and 18S ribosomal RNA (Mm03928990_g1). Primers were purchased from Life Technologies Corp. The mRNA levels of ATF4, DDIT3, GDF15, and FGF21 were standardized to the level of 18S ribosomal RNA.

### Complex IV activity assay.

Complex IV activity in the brain, liver, and muscle was measured in isolated mitochondria using a complex activity kit (ab109911, Abcam) following the manufacturer’s instructions. Mitochondria isolation buffer consisting of 70 mM sucrose, 210 mM mannitol, 5 mM HEPES, and 1 mM EGTA was used to isolate mitochondria from fresh samples.

### Measurements of H_2_S levels in brain, liver, muscle, feces, and plasma.

We used the H_2_S-specific fluorescent probe HSip-1 (synthesized and provided by the laboratory of Kenjiro Hanaoka, Graduate School of Pharmaceutical Sciences, Keio University, Tokyo, Japan) to measure H_2_S levels, as reported previously (13). After venous blood was drawn from anesthetized mice, mice were transcardially perfused with ice-cold PBS. Feces were collected from the ileocecum. Brain, liver, muscle, feces, and plasma were snap-frozen in liquid nitrogen and stored at –80°C until measurement. Brain, liver, and muscle were homogenized in 10 μM HSip-1 solution in HBSS (Thermo Fisher Scientific). Because the H_2_S levels in feces were expected to be higher than those in host tissues, feces were homogenized in a higher concentration (100 μM) of HSip-1 solution. Lysates were centrifuged at 14,000*g* for 5 minutes at 4°C after incubation at room temperature in the dark for 20 minutes. Plasma was separated from red blood cells by centrifugation at 4,000*g* at 4°C for 1 minute. Plasma was mixed with 5 μM HSip-1 solution and incubated at room temperature in the dark for 20 minutes. The fluorescence intensities were measured using a Varioskan LUX Multimode Microplate Reader (Thermo Fisher Scientific) at the wavelength of λ_ex_/λ_em_ = 491 nm/516 nm. The fluorescence intensities were normalized by the weight of the tissues.

### Standard curve for H_2_S levels assessed by HSip-1.

To construct a standard curve for the tissue H_2_S levels assessed by HSip-1, we measured fluorescence intensities when homogenates of brain, liver, muscle, and feces or plasma were incubated with vehicle or Na_2_S (15). Briefly, frozen tissues were homogenized in 10 μL/mg sample weight (brain) or 40 μL/mg sample weight (liver, muscle, and feces) of HBSS with HSip-1 at 10 μM or 100 μM, respectively. Frozen plasma (50 μL) was thawed and mixed in 450 μL of HBSS with HSip-1 at 5 μM. Solutions were incubated at room temperature in the dark for 20 minutes and centrifuged at 14,000*g* at 4°C for 5 minutes to obtain sample supernatants. Ninety microliters of sample supernatants were added in a 96-well plate before preparation of Na_2_S solution. Because the half-life of H_2_S is very short in a buffer with physiological pH (e.g., pH 7.4), we dissolved Na_2_S (sodium sulfide nonahydrate, Sigma-Aldrich) in Tris-HCl buffer (25 mM, pH 9.4) (49). Tris-HCl buffer was deoxygenated using nitrogen bubbles before preparation of Na_2_S solution. Na_2_S was quickly dissolved in Tris-HCl at 1 mM and serially diluted to 1, 10, or 100 μM in Tris-HCl. Ten microliters of Na_2_S solution at 0, 0.1, 1, 10, or 100 μM was mixed with sample supernatants in a 96-well plate at 1.5 minutes after the initiation of dissolution of Na_2_S. The 96-well plate was incubated at room temperature in the dark for 20 minutes. The fluorescence intensities were measured using a microplate reader at the wavelength of λ_ex_/λ_em_ = 491 nm/516 nm.

### Thiosulfate measurement.

The level of thiosulfate in tissues was measured by LC-MS/MS. Tissues were homogenized with ice-cold buffer [70% methanol/30 mM acetate buffer (pH 6.5) containing 5 mM β-(4-hydrocyphenyl)ethyl iodoacetamide (HPE-IAM)] for 5 minutes. Tissue homogenates were then incubated for 20 minutes at 37°C in the dark, followed by centrifugation for 10 minutes at 14,000*g* at 4°C. Supernatant (135 μL) was collected and mixed with 15 μL of 10% formic acid. Pellets were resuspended with 0.1% SDS in PBS by sonication, and then protein concentration was measured by bicinchoninic acid assay. LC-MS/MS was performed as described previously (48). Samples were subjected to the UPLC system with a Hypersil Gold C-18 (100 × 2.1 mm, 3.0 μm; Thermo Fisher Scientific) column. Then, samples were eluted by a linear methanol gradient of the mobile phase (0%–90%, 15 minutes) in the presence of 0.1% formic acid at a flow rate of 0.2 mL/min at 40°C. The results were analyzed using Compound Discoverer 3.3 software (Thermo Fisher Scientific).

### Metabolomic analysis to evaluate NADH-reductive stress.

Blood was collected by cardiac puncture and immediately transferred to a K2 EDTA additive blood collection tube (BD365974, BD). The sample was centrifuged at 3,000*g*, 4°C, for 3 minutes. Supernatant was transferred to a new collection tube, snap-frozen, and stored at –80°C until it was used for the analysis. The method used for absolute quantification of metabolites has been detailed previously (24). Briefly, 30 μL of plasma was mixed with 137 μL of extraction solvent of ice-cold acetonitrile containing isotope internal standards (^13^C_6_-glucose, d_3_-lactate, ^13^C_3_-pyruvate, d_3_-α-hydroxybutyrate, ^13^C_2_-β-hydroxybutyrate, ^13^C_3_-alanine, and ^13^C_4_-2-ketobutyrate). The mixture was vortexed and incubated on ice for 30 minutes. After centrifugation at 21,000*g* for 20 minutes at 4°C, 75 μL of supernatants were transferred to autosampler glass vials for LC-MS/MS analysis. Calibration curve samples were generated for each metabolite and were processed in the same manner. Quantitative analysis was performed using a Dionex Ultimate 3000 UHPLC system (Thermo Fisher Scientific) with a Waters XBridge amide column (2.1 × 100 mm × 2.5 μm) maintained at 27°C. Mobile phase A was 5% acetonitrile with 20 mM ammonium acetate (pH 9), and mobile phase B was acetonitrile. The LC gradient conditions at a flow rate of 0.220 mL/min were: 0 minutes 85% B, 0.5 minutes 85% B, 9 minutes 35% B, 11 minutes 2% B, 12 minutes 2% B, 13.5 minutes 85% B, 14.6 minutes 85% B, 15 minutes 85% B with 0.420 mL/min to 18 minutes. The elute was delivered into a Q-Exactive Plus Orbitrap mass spectrometer (Thermo Fisher Scientific) with heated electrospray ionization probe operating in polarity switching mode. MS parameters were: sheath gas flow 50, auxiliary gas flow 10, sweep gas flow 2, spray voltage 2.50 kV in negative and 3.8 kV in positive, capillary temperature 310°C, S-lens RF level −50, and auxiliary gas heater temperature 370°C. Data acquisition was performed using Xcalibur software (Thermo Fisher Scientific) in the range of 70–1,000 *m*/*z*, resolution 70,000, automatic gain control target 3 × 10^6^, and maximum injection time of 80 milliseconds. TraceFinder 4.1 was used for data processing with a 5 ppm mass tolerance. The results were quantified by comparison of the integrated peaks against a standard curve.

### Measurement of blood lactate.

Blood lactate was measured using an electrochemical lactate oxidase biosensor (Lactate Plus, Nova Biomedical). Blood was collected by tail snip at postnatal ages 30, 40, and 50 days.

### Measurement of acylcarnitine.

Venous blood was collected and transferred to EDTA-treated plastic tubes. Plasma was obtained by centrifugation at 1,500*g* at 4°C for 2 minutes. Acylcarnitine level was obtained by combining of data derived from LC-MS/MS and GC-MS/MS. Plasma was homogenized in methanol (100 mg/mL) via a ShakeMaster Auto (Biomedical Science Inc.), and samples were centrifuged at 15,000*g* for 5 minutes. Supernatants were applied to each analysis.

For LC-MS/MS, 100 μL of supernatant was mixed with 25 μL of 100 mM ammonium formate. The mixture was vortexed and centrifuged at 15,000 rpm for 5 minutes. Then, 2 μL of supernatant was injected into a LC-MS/MS system consisting of the UHPLC Nexera LC (Shimadzu) and a 5500 QTRAP mass spectrometer (AB Sciex Pte. Ltd.). Chromatographic separation was performed via the ZIC-cHILIC column (2.1 × 100 mm, 3 μm; Merck Millipore), with the column temperature at 30°C, by gradient elution of mobile phase A (10 mM ammonium formate aqueous solution) and mobile phase B (acetonitrile). The gradient program was as follows: 0–1.5 minutes, 97% B; 1.5–5 minutes, 97%–75% B; 5–7 minutes, 75% B; 7–10 minutes, 75%–40% B; 10–12 minutes, 40% B; 12–13 minutes, 40%–10% B; 13–16 minutes, 10% B; and 16–25 minutes, 97% B, with a flow rate of 0.4 mL/min. The LC eluate was directly introduced into a turbo-spray ionization source with simultaneous polarity switching, whose ionization parameters appear in Supplemental Table 1. Multiple reaction monitoring (MRM) was used to detect metabolites, with the detection conditions set on the basis of information in a previous report (50). LC-MS/MS data were processed via MultiQuant 3.0 (AB Sciex Pte. Ltd.).

For GC-MS/MS, 10 μL of the homogenized supernatant was dried using nitrogen stream, after which it was processed by 2-step reactions: oximation and trimethylsilylation. Oximation was achieved by addition of 25 μL of *O*-methylhydroxylammonium chloride in pyrimidine (15 mg/mL) and incubation of the sample at 40°C for 60 minutes. Then, for trimethylsilylation, 25 μL of *N*,*O*-bis(trimethylsilyl)trifluoroacetamide with 1% trimethylchlorosilane was added to the reaction solution, and incubation proceeded at 60°C for 60 minutes. The reaction mixture, 1 μL, was injected into an Agilent 7890B series GC system in split injection mode (10:1, vol/vol) via a GC injector 80 autosampler (Agilent Technologies Inc.). GC separation was performed with a J&W Scientific DM-5MS-DG column (30 m × 0.25 mm inner diameter, film thickness = 0.25 μm; Agilent Technologies Inc.) with a temperature gradient that rose at 10°C/min from 60°C to 325°C, and with a constant helium gas flow at 1 mL/min. The elution was ionized by means of electron impact ionization (70 eV) with an ion source temperature of 280°C, and it was introduced into an Agilent 7010B triple-quadrupole mass spectrometer. Each target molecule was detected by MRM, and its peak area was calculated by MassHunter software (Agilent Technologies Inc.).

### Brain MRI.

Brain MRI scans of three 8-week-old Sqor^ΔN/ΔN^ mice and one control mouse were performed as previously reported (32, 51). Mice were continuously anesthetized with 0.5%–1.0% isoflurane in room air during the imaging procedure. T2-weighted RARE (rapid acquisition of refocused echoes) MRI images were acquired on a 4.7-T small-animal scanner (PharmaScan, Bruker) with the following parameters: RARE factor, 10; echo time, 60 milliseconds; repetition time, 6,000 milliseconds; averages: 8,192 × 192 × 24 image matrix with a voxel size of 0.130 × 0.130 × 0.7 mm. The locations of brain lesions were determined based on the Allen brain atlas (https://atlas.brain-map.org/atlas?atlas=1; accessed February 15, 2023).

### Brain tissue PO_2_ measurement.

Brain tissue PO_2_ was measured at postnatal age 40 days as previously reported (32). Mice were anesthetized with 0.5%–1.0% isoflurane in room air during the measurement. Body temperature was maintained at 37°C for control mice using TCAT-2LV Temperature Controller (Physitemp Instruments). Because the body temperature of Sqor^ΔN/ΔN^ mice decreased with time and was approximately 34°C at postnatal age 40 days (Figure 1C), the body temperature of Sqor^ΔN/ΔN^ mice was maintained at 34°C during the procedure. Mice were placed in a prone position, and the head was stabilized using a stereotaxic frame (ASI Instruments). After skin incision, holes were drilled in the skull with a micro-drill (MD-1200, Braintree Scientific), and an optical PO_2_ probe (OxyLab, Oxford Optronics) was inserted at target locations. Brain tissue PO_2_ was measured at midbrain reticular nucleus and caudoputamen, and the average values at these 2 locations were compared between Sqor^ΔN/ΔN^ mice and control mice. The coordinates for the midbrain reticular nucleus were mediolateral [ML] = 0.70 mm, anteroposterior [AP] = –2.90 mm, and dorsoventral [DV] = –3.50 mm from the bregma, while the coordinates for the caudoputamen were ML = 1.70 mm, AP = 0.25 mm, and DV = –3.40 mm from the bregma.

### Statistics.

Data are presented as mean ± SD (parametric data) or medians with interquartile range (non-parametric data). Descriptive statistics were used to describe the study population. We used Shapiro-Wilk test and Q-Q plots to assess the normality of the data. Parametric data were analyzed using unpaired 2-tailed *t* test to compare 2 groups, and 1-way ANOVA with Dunnett’s multiple-comparison test to compare 3 groups. Non-parametric data were analyzed using Mann-Whitney test to compare 2 groups, and Kruskal-Wallis test with Dunn’s multiple-comparison test to compare 3 groups. Mixed-effects analysis with Dunnett’s multiple-comparison test was performed to compare the blood lactate levels at postnatal ages 30, 40, and 50 days in Sqor^ΔN/ΔN^ mice. A 2-tailed *t* test with Welch’s correction was performed to compare C4 acylcarnitines between Sqor^ΔN/ΔN^ mice and control mice. Survival data were visualized using a Kaplan-Meier survival plot, and the log-rank test was used to compare survival curves. Significance was considered at the level of *P* less than 0.05. Statistical analyses were conducted using Prism 9.2.0 (GraphPad Software Inc.).

### Study approval.

All procedures were performed in accordance with animal protocols approved by Massachusetts General Hospital Institutional Animal Care and Use Committee and the National Research Council’s *Guide for the Care and Use of Laboratory Animals* (National Academies Press, 2011).

### Data availability.

Values for all data points in graphs are reported in the Supporting Data Values file.

## Author contributions

EK and FI designed research. EK, KS, EM, M Morita, M Miranda, YM, CS, RS, and FD performed experiments. EK, KS, EM, M Morita, M Miranda, YM, CS, RS, FD, DBB, TA, and FI analyzed data. EK, DBB, and FI wrote the original draft. EK, KS, DBB, and FI wrote the revised paper. The order of the 3 co–first authors (EK, KS, and EM) was determined based on the intellectual contribution of each author. All authors contributed to the article and approved the submitted version.

## Supplementary Material

Supplemental data

Supporting data values

## Version 1 06/13/2024

In-Press Preview

## Version 2 08/01/2024

Electronic publication
