# Supplementary material for: Exclusion of sulfide:quinone oxidoreductase from mitochondria causes Leigh-like disease in mice by impairing sulfide metabolism
Source: J Clin Invest. 2024 Jun 13;134(15):e170994. doi: 10.1172/JCI170994 (PMC11290971; doi:10.1172/JCI170994)
Supplement: Supplemental data [file jci-134-170994-s124.pdf]

Supplemental Figure 1

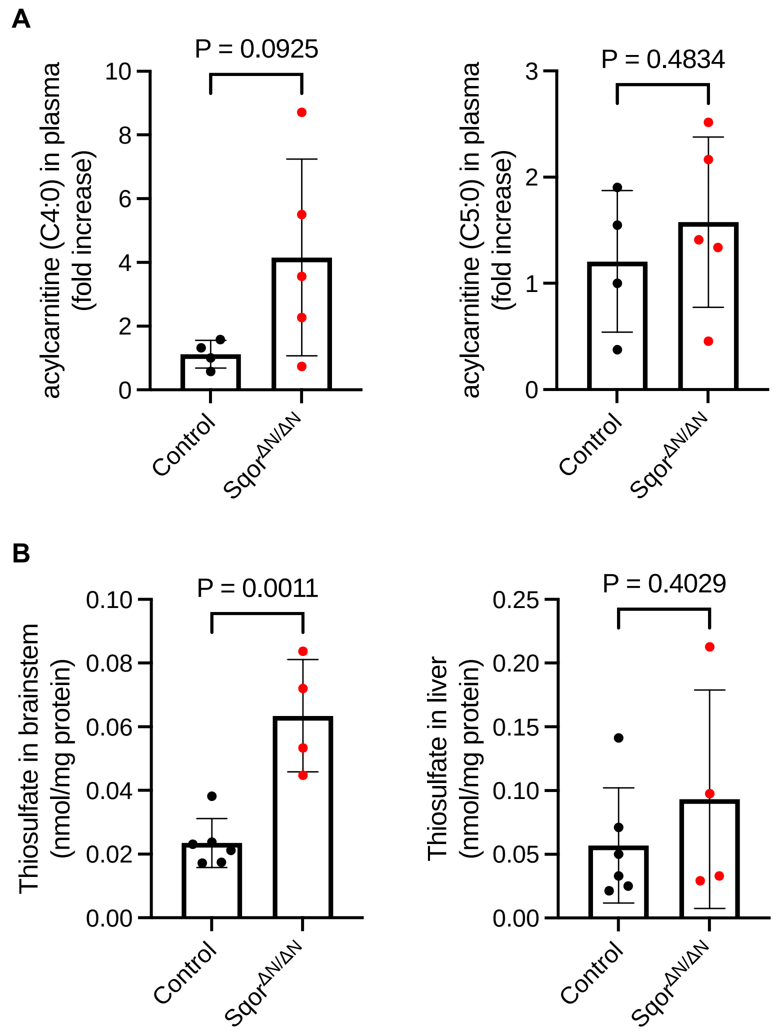

Figure S1. Measurement of blood C4 and C5 acylcarnitines and thiosulfate in brainstem and liver of Sqor$^{\Delta N/\Delta N}$ mice.

(A) C4 and C5 acylcarnitines in plasma of Sqor$^{\Delta N/\Delta N}$ mice were measured using LC-MS/MS and GC-MS/MS. Data were analyzed using t-test with Welch's correction for C4 acylcarnitine and t-test for C5 acylcarnitine. n = 4 to 5 mice for each group. Data are presented as means with standard deviation. (B) Thiosulfate in the brainstem and liver of Sqor$^{\Delta N/\Delta N}$ mouse was measured using LC-MS/MS. Data were analyzed using t-test. n = 4 to 6 mice for each group. Data are presented as means with standard deviation.

Abbreviations: DMSO, dimethyl sulfoxide; GC-MS/MS, gas chromatography-tandem mass spectrometry; LC-MS/MS, liquid chromatography-tandem mass spectrometry.

Supplemental Figure 2

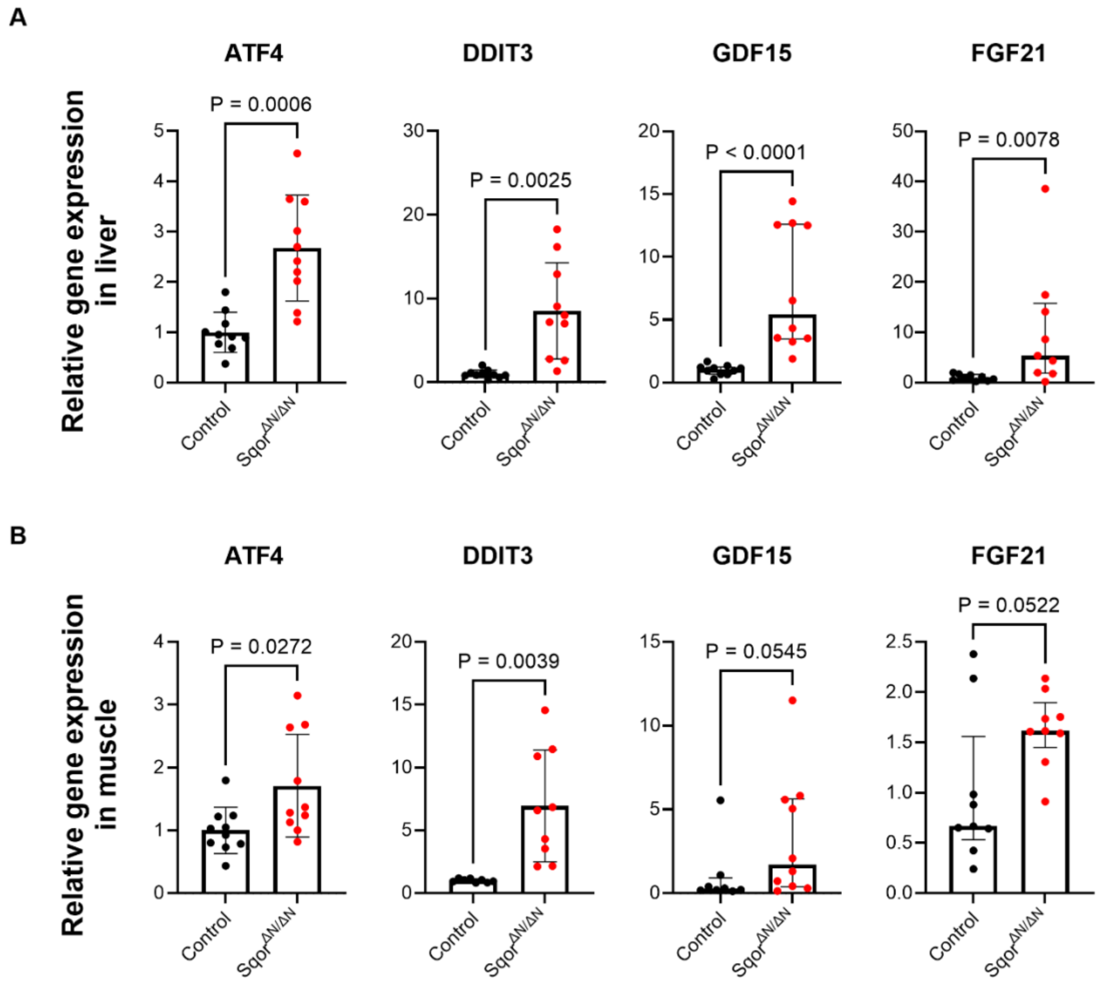

Figure S2. Integrated stress response was triggered by mutations in Sqor$^{\Delta N/\Delta N}$ mouse.

The mRNA levels of ATF4, DDIT3, GDF15, and FGF21 were measured using liver (A) and muscle (B) of Sqor$^{\Delta N/\Delta N}$ mice. Data were analyzed using t-test for ATF4 and DDIT3 and Mann-Whitney test for GDF15 and FGF21. n = 8 to 10 mice for each group. Data are presented as means with standard deviation for ATF4 and DDIT3, and as medians with interquartile range for GDF15 and FGF21. Abbreviations: ATF4, activating transcription factor 4; DDIT3, DNA damage-inducible transcript three protein; FGF21, fibroblast growth factor 21; GDF15, growth/differentiation factor 15; mRNA, messenger RNA.

Supplemental Figure 3

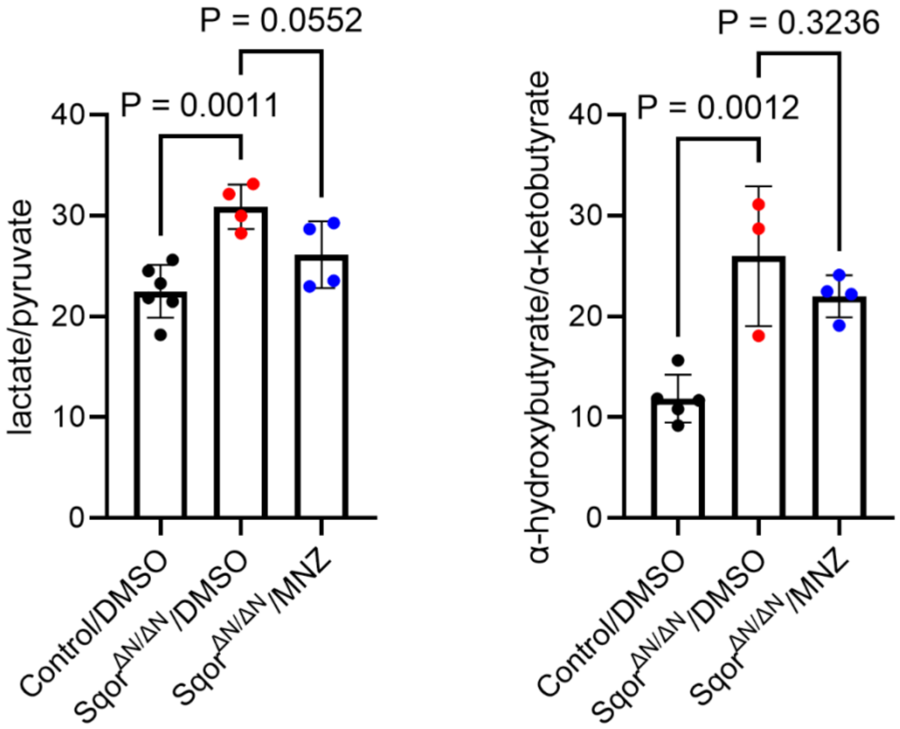

Figure S3. NADH-reductive stress was induced by mutations in Sqor$^{ΔN/ΔN}$ mice.

The concentrations of lactate, pyruvate, α-hydroxybutyrate, and α-ketobutyrate in plasma of Sqor$^{ΔN/ΔN}$ mice were measured by LC-MS/MS, and lactate/pyruvate ratio and α-hydroxybutyrate/α-ketobutyrate ratio were calculated. Data were analyzed using one-way ANOVA with Dunnett's multiple comparisons test. n = 3 to 6 mice for each group. Data are presented as means with standard deviation. Abbreviations: DMSO, dimethyl sulfoxide; LC-MS/MS, liquid chromatography-tandem mass spectrometry; MNZ, metronidazole.

Supplemental Figure 4

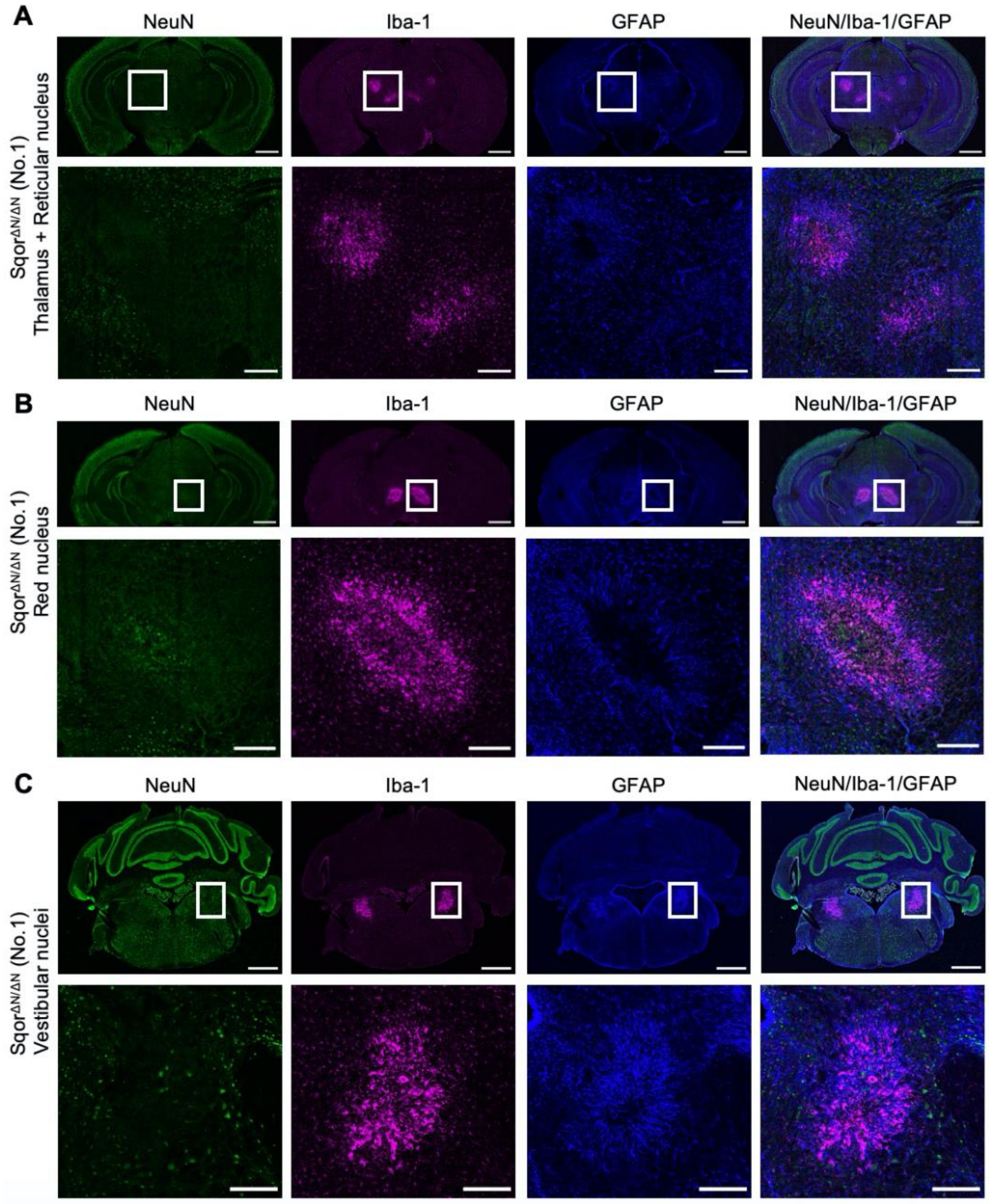

Figure S4. Results of NeuN, Iba-1, and GFAP immunohistochemical staining in Sqor$^{\Delta N/\Delta N}$ mouse No.1.

The portions of the thalamus and reticular nucleus (A), red nucleus (B), and vestibular nuclei (C) of Sqor$^{\Delta N/\Delta N}$ mouse No.1 that were indicated in the brain MRI scans were used for immunohistochemical staining. Staining for NeuN, Iba-1, and GFAP was performed to evaluate neurodegeneration and gliosis. An enlargement of the boxed regions is provided below each cross-section. Scale bar = 1000 μm

in low magnification images and 250 μm in high magnification images. Abbreviations: GFAP, glial fibrillary acidic protein; Iba-1, ionized calcium-binding adaptor molecule 1; NeuN, neuronal nuclei.

Supplemental Figure 5

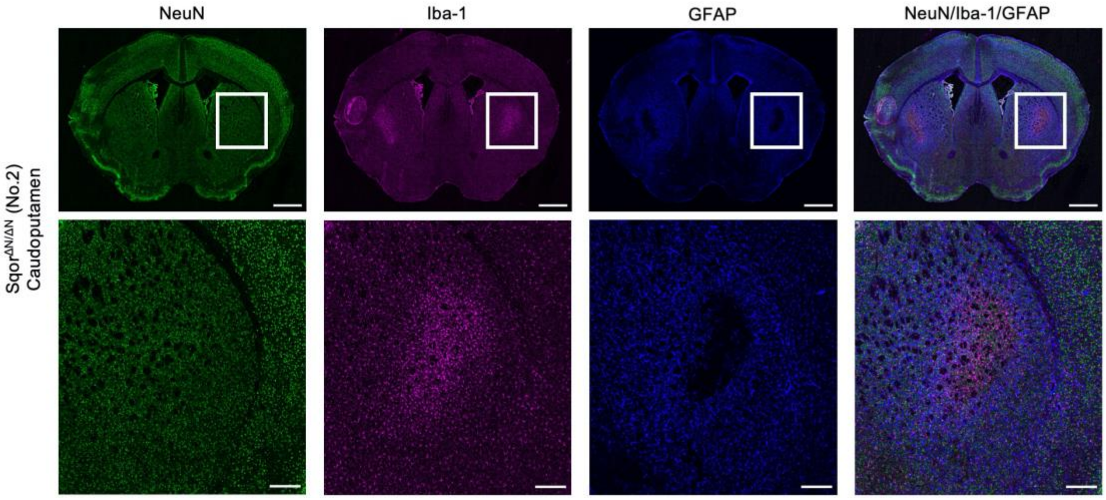

Figure S5. Results of NeuN, Iba-1, and GFAP immunohistochemical staining in Sqor$^{\Delta N/\Delta N}$ mouse No.2.

The portions of the caudoputamen of Sqor$^{\Delta N/\Delta N}$ mouse No.2 that were indicated in the brain MRI scans were used for immunohistochemical staining. Staining for NeuN, Iba-1, and GFAP was performed to evaluate neurodegeneration and gliosis. An enlargement of the boxed regions is provided below each cross-section. Scale bar = 1000 μm in low magnification images and 250 μm in high magnification images.

Abbreviations: GFAP, glial fibrillary acidic protein; Iba-1, ionized calcium-binding adaptor molecule 1; NeuN, neuronal nuclei.

Supplemental Figure 6

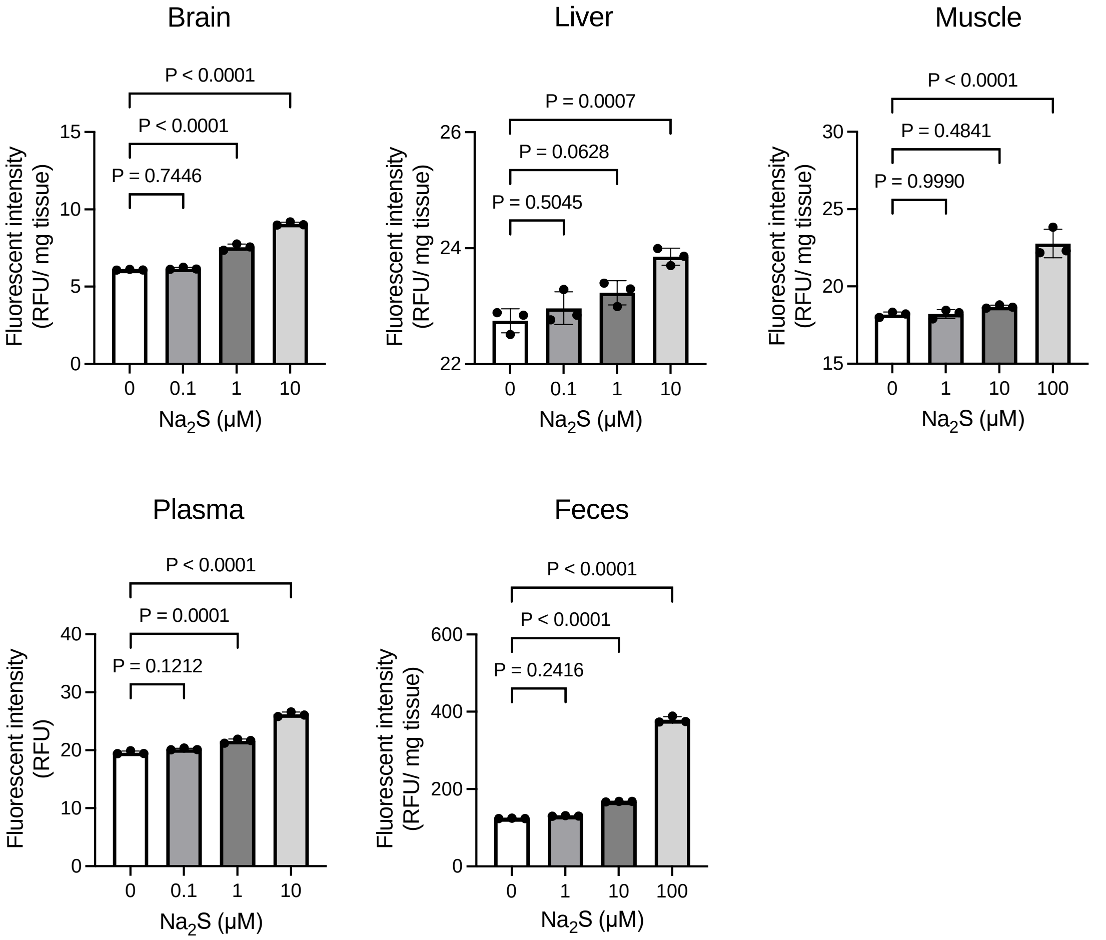

Figure S6. Dose-dependent increase of HSip-1 fluorescent intensities in response to exogenously added Na_2S.

Fluorescent intensities at the wavelength of λex/λem = 491 nm/516 nm in HSip-1 solution with brain, liver, muscle, plasma, or feces incubated with vehicle or Na_2S. Data were analyzed using one-way ANOVA with Dunnett's multiple comparisons test. n = 3 for each group. Data are presented as means with standard deviation.

Abbreviations: Na_2S, sodium sulfide; RFU, relative fluorescence unit.

Supplementary Table

**Parameter**		
Polarity	Positive	Negative
Ion spray voltage	4,300	-4,300
Curtain gas	40	40
Collision gas	12	12
Temperature	475	475
Ion source gas 1	30	30
Ion source gas 2	50	50

Supplementary Table. Parameters of turbo spray ionization
